# Effects of Saccharides from Arctium lappa L. Root on FeCl_3_-Induced Arterial Thrombosis via the ERK/NF-*κ*B Signaling Pathway

**DOI:** 10.1155/2020/7691352

**Published:** 2020-03-24

**Authors:** Tiantian Qiu, Hailun Zhou, Suying Li, Nuoya Tian, Zhe Li, Rui Wang, Pengyuan Sun, Jinyong Peng, Jianling Du, Xiaochi Ma, Yunpeng Diao, Li Lv, Li Wang, Hua Li

**Affiliations:** ^1^Department of Pharmacology, College of Pharmacy, Dalian Medical University, Dalian, Liaoning Province, China; ^2^Department of Endocrinology, First Affiliated Hospital of Dalian Medical University, Dalian, Liaoning Province, China; ^3^National-Local Joint Engineering Research Center for Drug-Research and Development (R & D) of Neurodegenerative Diseases, Dalian Medical University, Dalian, Liaoning Province, China

## Abstract

Saccharides from Arctium lappa. L. root (ALR-S) is a high-purity fructosaccharide separated from the medicinal plant Arctium lappa. L. root. These compounds showed many pharmacological effects in previous studies. In the present study, the antithrombotic effects of ALR-S in arterial thrombosis via inhibiting platelet adhesion and rebalancing thrombotic and antithrombotic factor expression and secretion were found in rats and human aortic endothelial cells (HAECs). This study also showed that inhibition of oxidative stress (OS), which is closely involved in the expression of coagulation- and thrombosis-related proteins, was involved in the antithrombotic effects of ALR-S. Furthermore, studies using FeCl_3_-treated HAECs showed that ALR-S induced the abovementioned effects at least partly by blocking the ERK/NF-*κ*B pathway. Moreover, U0126, a specific inhibitor of ERK, exhibited the same effects with ALR-S on a thrombotic process in FeCl_3_-injured HAECs, suggesting the thrombotic role of the ERK/NF-*κ*B pathway and the antithrombotic role of blocking the ERK/NF-*κ*B pathway by ALR-S. In conclusion, our study revealed that the ERK/NF-*κ*B pathway is a potential therapeutic target in arterial thrombosis and that ALR-S has good characteristics for the cure of arterial thrombosis via regulating the ERK/NF-*κ*B signaling pathway.

## 1. Introduction

Arterial thrombosis is one of the main underlying causes of cardiovascular disease. The endothelium is responsible for many functions, such as the control of vascular permeability and tone, maintenance of vessel integrity, regulation of vascular inflammation, and prevention of thrombosis [1, 2]. Endothelial dysfunction is the hallmark and indeed a predictor of cardiovascular disease. In atherosclerosis, diabetes, and other vascular pathology, oxidative stress signals activate vascular endothelial cells (VECs), inducing the expression of reactive oxygen species (ROS) and decreasing vessel endothelial integrity. VECs can synthesize and release antithrombotic factors, including prostacyclin (PGI_2_), tissue factor pathway inhibitor (TFPI), thrombomodulin (TM), and platelet endothelial cell adhesion molecule-1 (PECAM-1), which inhibit blood coagulation and platelet adhesion and aggregation and finally prevent thrombosis [3, 4].

PGI_2_ can relax blood vessels and inhibit platelet aggregation. The FXa-TFPI-FVIIa-TF tetramer was combined by TFPI with tissue factor (TF), then coagulated factors VII(FVII) and X(FX), thus inactivating tissue factor pathway and inhibiting coagulation reaction. TM can reversibly bind to the endothelial cell protein C receptor and reduce the level of free thrombin, thus preventing thrombin from cleaving fibrinogen or activating platelets. After endothelial cell injury, TM on the endothelial cell surface is released into the blood, which reduces the thrombin binding rate and the antithrombotic effect of endothelial cells [5]. Therefore, plasma TM content is a gold-standard indicator of endothelial cell injury. PECAM-1, synthesized by endothelial cells, is a key factor involved in platelet adhesion and aggregation. PECAM-1 can inhibit platelet aggregation and thrombosis by regulating the phosphorylation of intracytoplasmic signaling molecules to restrict communication of the activation response between cells. When endothelial cells are damaged, PECAM-1 is highly expressed, which initiates a negative feedback mechanism and inhibits thrombosis [6].

When VECs are damaged, they slough off, leading to exposure of subendothelial tissue and potentially the release of a large amount of TF. The TF complex with FVII in the blood activates the exogenous coagulation pathway to produce thrombin. Thrombin activates XIII and causes fibrin to form blood clots. In addition, when VECs are injured, a large amount of vWF is released to stimulate platelet activation and produce platelet aggregation. At the same time, activated platelets release TXA_2_, ADP, and other substances to further promote platelet adhesion, aggregation, and thrombosis.

After vascular injury, VECs, vascular smooth muscle cells, leukocytes, fibroblasts, and platelets are important sources of ROS, which can regulate platelet activation [7, 8], triggering a vicious circle that leads to thrombosis. NADPH oxidase is the main regulator in the antioxidant system in organisms [9–13]. NOX4 is the primary subtype of NADPH oxidase expressed in VECs and participates in the production of ROS in VECs [14]. If a large amount of ROS cannot be eliminated quickly from the body, the high ROS levels can activate NF-*κ*B, AP-1, and/or other signaling pathways through extracellular regulated protein kinase 1/2 (ERK1/2). These activation events cause lipid peroxidation, which injures VECs and promotes the overexpression of thrombosis factors, inflammatory factors, and adhesion factors. These factors can further injure VECs, cause thrombosis, and induce cardiovascular disease [15, 16].

In addition to treating thrombosis with antithrombotic agents, there is increasing interest in the use of natural food products and biologically active ingredients for the prevention and treatment of thrombosis. ALR-S contain fructosaccharides that can be separated at high purity. These polysaccharides have many pharmacological effects, such as anti-inflammatory and immune regulatory activity [17, 18]; antifatigue [19], anticancer [20], and hypolipidemic effects [21]; and protection against alcohol damage [22]. They also have obvious antioxidant effects in vivo and in vitro [23].

In this study, we used a FeCl_3_-induced rat arterial thrombosis model and a human aortic endothelial cell (HAEC) injury model to explore whether ALR-S can inhibit excessive OS and regulate the expression of thrombosis-associated factors through the ERK/NF-*κ*B signaling pathway, leading to antithrombotic effects.

## 2. Materials and Methods

### 2.1. Preparation of ALR-S

ALR-S was extracted and purified from Arctium lappa L. root in our laboratory. The dry ALR powder was macerated in 80% alcohol at 60°C for 6 h and refluxed in aether petrolei at 45°C for 1.5 h to remove soluble contaminants. The residues were vacuum-dried and were macerated in distilled water at 95°C for 3 h; then, the filtrate was concentrated. The Sevag method was used to remove proteins [24]. The deproteinized solution was precipitated by 75% alcohol for more than 12 h, then centrifuged at 5000 rpm, 15 min, and the precipitation was extracted in water. Then, the polysaccharide precipitation was collected, washed with anhydrous alcohol and acetone, and freeze-dried to produce the crude ALR-S. The crude ALR-S was filtered twice by Sephadex G-50 column chromatography to obtain the purified ALR-S. Purity and molecular weight of ALR-S were detected by a high-performance gel permeation chromatography (HP-GPC) method. Infrared spectroscopy and liquid chromatography-mass spectrometry (LC-MS) were used for detecting fructose configuration and content. The results showed that the average molecular weight was 2135 Da and the purity was 97.7%. The content of fructose was about 86%, mainly in the form of fructofuranose.

### 2.2. Animals and Treatment

Male Sprague-Dawley (SD) rats weighing 180 g to 220 g were purchased from the Animal Center of Dalian Medical University (Dalian, China). The rats were housed under 12 h light/12 h dark cycles at 21 ± 3°C and 55 ± 5% relative humidity.

Sixty male SD rats were randomly divided into six groups (*n* = 10): (1) control group (normal saline, iv), (2) FeCl_3_-induced arterial thrombosis model group (model group, normal saline, iv), (3) low-dose ALR-S group (ALR-S, 1 mg/kg, iv), (4) middle-dose ALR-S group (ALR-S, 2 mg/kg, iv), (5) high-dose ALR-S group (ALR-S, 4 mg/kg, iv), and (5) heparin group (heparin, 150 U/kg, iv).

All the groups of male SD rats were anesthetized with 20% urethane (50 mg/kg, i.p.). Different concentrations of ALR-S, heparin, or normal saline were injected, respectively, into the right femoral vein at 2 ml/kg for each group. Twenty minutes later, the common carotid artery thrombosis model was prepared according to Kurz et al.'s method [25, 26]. In all groups, a 1 cm portion of the right common carotid artery was exposed by careful blunt dissection after a midline cervical incision. A small piece of plastic film was placed underneath the artery to separate it from surrounding tissues. A small piece of filter paper (5 mm × 5 mm) was soaked in 10 *μ*l solution of 25% FeCl_3_ and then applied for 5 min on the right common carotid artery to induce thrombosis. Then, the filter paper was removed, and the surgical site was rinsed with sterile saline to remove excess FeCl_3_. The control group underwent the same surgery, but FeCl_3_ was replaced with normal saline.

After 10 minutes, blood samples were collected from the rats via the abdominal aorta. The blood was anticoagulated by 3.8% sodium citrate and then centrifuged (4°C, 3000 rpm, 10 min) to obtain plasma. The supernatant (plasma) was stored at -80°C.

The same length of the right common carotid artery segment containing the thrombus of all rats was dissected and weighed. Then, the arterial thrombus were carefully removed and the blood vessels were stored at -80°C.

### 2.3. Cell Culture and Treatment

Human aortic endothelial cells (HAECs) were purchased from Shanghai Institute of Biochemistry and Cell Biology (Shanghai, China) and cultured in DMEM (Gibco, Gaithersburg, USA) with 10% (*v*/*v*) fetal bovine serum (FBS, ExCell Bio, South America), 4.5 g/l glucose, 4.0 mM L-glutamine, 100 U/ml penicillin, and 0.1 mg/ml streptomycin at 37°C in an incubator with 5% CO_2_.

HAECs were divided into five groups: (1) control group (serum-free DMEM), (2) model group (0.4 mmol/l FeCl_3_), (3) low-dose ALR-S group (0.1 mg/ml ALR-S), (4) middle-dose ALR-S group (0.2 mg/ml ALR-S), and (5) high-dose ALR-S group (0.4 mg/ml ALR-S). HAECs (1 × 10^5^) were seeded on six-well plates and pretreated at 90% confluence with different concentrations of ALR-S or vehicle for 4 h and then exposed to 0.4 mmol/l FeCl_3_ for 2 h.

For ERK1/2 inhibition study, HAECs were divided into five groups: (1) control group (serum-free DMEM), (2) model group (0.4 mmol/l FeCl_3_), (3) ALR-S (0.4 mg/ml ALR-S), and (4) U0126 (10 *μ*mol/l U0126). HAECs (1 × 10^5^) were seeded on six-well plates and pretreated at 90% confluence with ALR-S or U0126 for 4 h and then stimulated with 0.4 mmol/l FeCl_3_ for 2 h.

### 2.4. Biochemical Analysis

Plasma malondialdehyde (MDA), superoxide dismutase (SOD), lactate dehydrogenase (LDH), hydrogen peroxide (H_2_O_2_), and cellular SOD, LDH, glutathione (GSH), and nitric oxide (NO) in culture medium in HAECs were detected with kits (Nanjing Jiancheng Institute of Biotechnology, Nanjing, China; Beyotime Biotechnology, Shanghai, China.).

### 2.5. Measurement of Platelet ROS

Platelet-rich plasma (PRP), platelet-poor plasma (PPP), and washed platelets were prepared as described previously [27, 28]. Briefly, the blood was centrifuged (room temperature, 300 ×g, 15 min) to prepare PRP. The blood was then centrifuged (room temperature, 1500 ×g, 5 min) to prepare the platelet-poor plasma (PPP).

Platelets ROS were detected using the 2,7-dichlorodihydrofluorescein diacetate (DCFH-DA) probe according to the instructions in the detection kit (Beyotime Institute of Biotechnology, China). Briefly, the platelets were incubated with the probe (5 *μ*M) for 30 min at 37°C in the dark. The relative fluorescence units of the platelets were measured by a fluorescence microplate reader (Thermo Scientific, USA) using excitation at 488 nm and emission at 525 nm.

### 2.6. Measurement of Intracellular ROS

Intracellular ROS in HAECs were detected with the same detection kit as Section 2.5. Each group of cells was collected and incubated with the DCFH-DA probe (10 *μ*M) for 30 min at 37°C in the dark. Fluorescence in each group was measured by flow cytometry (Becton Dickinson, USA) using excitation at 488 nm and emission at 525 nm. Cell fluorescence was also observed by inverted fluorescence microscopy.

### 2.7. Western Blot Analysis

A commercial protein isolation kit (KeyGEN Biotech, Nanjing, China) was used for harvesting and extracting the nuclear proteins, cytoplasmic proteins, and total protein in tissues or HAECs. Equal loading quantities (15-30 *μ*g) of protein were separated by 8%~15% sodium dodecyl sulfate-polyacrylamide gel electrophoresis (SDS-PAGE). Then, the target proteins were transferred to polyvinylidene difluoride (PVDF) membranes (Millipore, Bedford, MA, USA). The membranes were blocked with 5% milk for 60~90 min at room temperature and were incubated at 4°C overnight accordingly with the primary antibodies specific for TM (Bioss Antibodies, Beijing, China), TFPI (Elabscience, Wuhan, China), TF, FPA (Abcam, Cambridge, Britain), PECAM-1, p-ERK (Cell Signaling Technology, Boston, USA), ERK, NOX4, P65, lamin B, and GAPDH (Proteintech Group, Wuhan, China).

On the second day, the appropriate secondary antibodies were incubated at room temperature for 4 h. The target proteins were exposed to chemiluminescence plus reagents (BioTools, USA). Emitted light was measured by a multispectral imaging system (Upland, CA), and a Gel-Pro Analyzer (Version 4.0, Media Cybernetics, Rockville, USA) was used for analyzing bands of each group.

### 2.8. Enzyme-Linked Immunosorbent Assay (ELISA) Analysis

The supernatant was obtained from centrifuged rat plasma (4°C, 3000 rpm, 5 min) or from cell culture medium (4°C, 3000 rpm, 20 min). The contents of TXB_2_, 6-keto-PGF1*α*, vWF, and PECAM-1 in the supernatant were detected according to the instructions of the ELISA kits (Dongge Company, Beijing, China). The OD values were measured at 450 nm using a microplate reader (Thermo Scientific, USA). The contents of TXB_2_, 6-keto-PGF1*α*, vWF, and PECAM-1 were calculated according to the standard curve.

### 2.9. Transmission Electron Microscopy (TEM) Analysis

HAECs were washed with PBS, collected by centrifugation (room temperature, 2500 rpm, 10 min), and placed in 2.5% glutaraldehyde overnight at 4°C. The cells were rinsed three times with 0.1 M phosphate buffer for 15 min each, fixed for 1 hour with 1% osmium acid, and rinsed again as before. The cells were blocked with 1% uranium acetate for 2 hours and then dehydrated with an acetone gradient (50%, 70%, 80%, 90%, and 100%) for 15 minutes at each concentration and twice with 100% acetone for 10 minutes each. The cells were immersed consecutively in the following solutions: acetone : embedding solution (1 : 1) for 2 hours at 37°C; acetone : embedding solution (1 : 4) overnight at 37°C; and pure embedding solution for 2 hours at 45°C. Finally, the blocks of cells were sectioned and observed.

### 2.10. Platelet Adhesion Test

PRP, PPP, and washed platelets were prepared as described in Section 2.5. Washed platelets were suspended in PPP, and the counts were adjusted to 2 × 10^8^ platelets/ml.

Platelet adhesion test was performed as described previously [29]. HAECs were cultured and treated with different factors as described in Section 2.3. The medium was discarded, and cells were cocultured with PRP or PPP labeled with a 10 *μ*M BCECF probe (Beyotime Institute of Biotechnology, China) for 1 h. The fluorescence microplate reader (Thermo Scientific, USA) was used for detecting fluorescence in each group.

### 2.11. Statistical Analysis

Statistical analysis was performed by IBM SPSS 21.0 (Chicago, USA). All values are presented as the mean ± SD. *T*-test, one-way ANOVA, and the Student-Newman-Keuls (SNK) method were used for data analysis. *p* < 0.05 referred that the difference was statistically significant.

## 3. Results

### 3.1. ALR-S Inhibited FeCl_3_-Induced Common Carotid Artery Thrombosis in Rats

**Figure 1:**
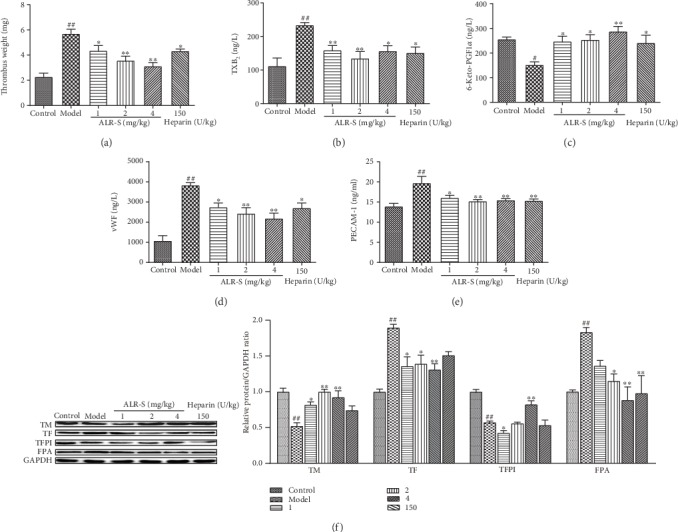
The effects of ALR-S on FeCl_3_-induced common carotid artery thrombosis in rats. Different doses of ALR-S (1, 2, and 4 mg/kg), heparin, or normal saline were injected into the right femoral vein at 2 ml/kg 20 minutes before initiating injury with 25%FeCl_3_. (a) Arterial thrombus weight. (b, c) Plasma TXB_2_ and 6-keto-PGF_1*α*_ contents, *n* = 10. (d, e) Plasma vWF and PECAM-1 contents, *n* = 10. (f) Protein expression level of TM, TF, TFPI, and FPA in the carotid artery vascular wall, *n* = 3. ^#^*p* < 0.05 and ^##^*p* < 0.01 compared to the control group; ^∗^*p* < 0.05 and ^∗∗^*p* < 0.01 compared to the model group.

Compared with the control group, the model group showed a significant increase in thrombus weight (Figure 1(a)). The ALR-S and heparin groups showed a significant decrease in thrombus weight compared with the model group.

The imbalance between thrombosis-relative regulatory factors, including TXB_2_, 6-keto-prostaglandin F_1*α*_ (6-keto-PGF_1*α*_), vWF, PECAM-1, TM, TFPI, TF, and FPA, was significantly improved by different doses of ALR-S (Figures 1(b)–1(f)) compared with the model group.

### 3.2. The Antioxidative Effects of ALR-S on FeCl_3_-Induced Common Carotid Artery Thrombosis in Rats

**Figure 2:**
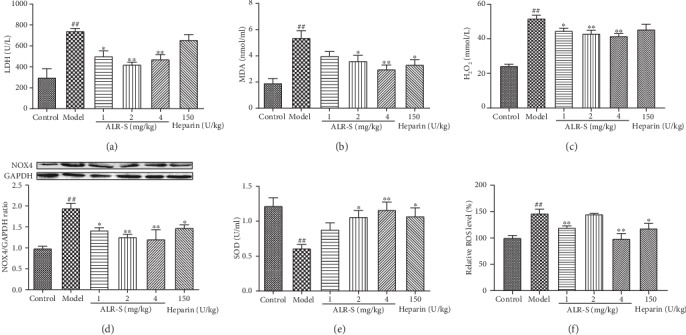
The antioxidant effect of ALR-S in FeCl_3_-induced common carotid artery thrombosis in rats. Different doses of ALR-S (1, 2, and 4 mg/kg), heparin, or normal saline were injected into the right femoral vein at 2 ml/kg 20 minutes before initiating injury with FeCl_3_. (a) LDH levels in rat plasma, *n* = 6. (b) MDA levels in rat plasma, *n* = 6. (c) H_2_O_2_ levels in rat plasma, *n* = 6. (d) Protein expression levels of NOX4 in the carotid artery vascular wall, *n* = 3. (e) SOD activities in rat plasma, *n* = 6. (f) The relative ROS levels in rat platelet, *n* = 6. ^##^*p* < 0.01 compared to the control group; ^∗∗^*p* < 0.01 compared to the model group.

One of the most important mechanisms of thrombosis was oxidative stress [30]. Antioxidants have shown potential antithrombotic effects in previous studies [31, 32]. Plasma LDH, MDA, and H_2_O_2_ levels in the model group were obviously increased compared with those in the control group, and the levels were obviously decreased in rats treated with ALR-S compared with model rats (Figures 2(a)–2(c)). The protein expression of NOX4, the primary subtype of NADPH oxidase, showed similar trends with those of plasma LDH, MDA, and H_2_O_2_ (Figure 2(d)). However, the plasma SOD levels in the model group were significantly decreased compared with those in the control group and increased in rats treated with ALR-S compared with the model group (Figure 2(e)). Platelet ROS levels showed similar trends with those of plasma LDH, MDA, and H_2_O_2_ (Figure 2(f)).

### 3.3. The Effects of ALR-S on Platelet Adhesion to FeCl_3_-Injured HAECs

**Figure 3:**
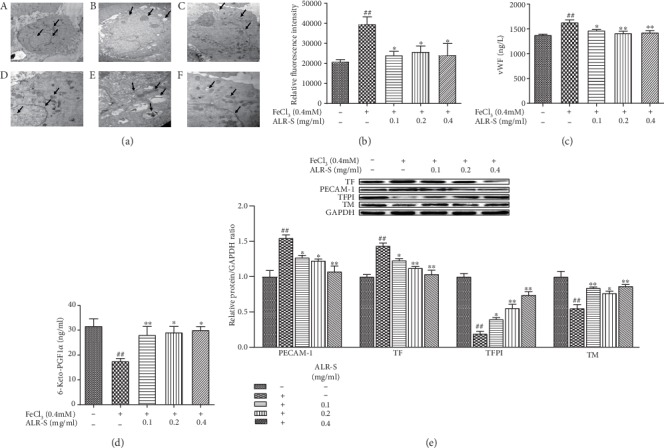
The protective effects of ALR-S in FeCl_3_-injured HAECs. HAECs were preincubated with different doses of ALR-S (0.1, 0.2, and 0.4 mg/ml) or serum-free medium for 4 h and then stimulated with 0.4 mM FeCl_3_ for 2 h. (a) TEM observation of HAEC morphology (A–C, 1 *μ*m; D–F, 500 nm). A and D, control group; B and E, FeCl_3_ group; C and F, ALR-S (0.4 mg/ml) group. (b) Platelet fluorescence intensity, *n* = 8. (c, d) The vWF and 6-keto-PGF_1*α*_ levels in HAEC culture medium, *n* = 8. (e) Protein expression level of PECAM-1, TFPI, TF, and TM in HAECs, *n* = 3. ^#^*p* < 0.05 and ^##^*p* < 0.01 compared to the control group; ^∗^*p* < 0.05 and ^∗∗^*p* < 0.01 compared to the FeCl_3_ group.

After incubation with FeCl_3_ for 2 h, the cells were observed by TEM (Figure 3(a)). The FeCl_3_ treatment group showed nucleus expansion or even fragmentation, mitochondrial swelling, the decrease or disappearance of mitochondrial cristae, and vacuolization, whereas ALS-OS pretreatment decreased nuclear fragmentation, mitochondrial swelling, and vacuolization and induced an obvious improvement in cell morphology.

The platelet adhesion to HAECs was determined by platelet fluorescence intensity. The FeCl_3_-treated group increased the platelet fluorescence intensity significantly compared to the control group, while the ALR-S pretreatment obviously reversed the FeCl_3_-induced platelet adhesion (Figure 3(b)).

The factors relative to platelet adhesion were determined. The medium levels of vWF were significantly increased in the FeCl_3_ group compared to the control group and decreased in the ALR-S groups (Figure 3(c)). The medium levels of 6-keto-PGF_1*α*_ were significantly decreased in the FeCl_3_ group and increased in the ALR-S groups (Figure 3(d)). The intracellular protein expression levels of PECAM-1 and TF were significantly increased in the FeCl_3_ group and decreased in the ALR-S groups. The intracellular protein expression levels of TFPI and TM were decreased in the FeCl_3_ group and increased in the ALR-S groups (Figure 3(e)).

### 3.4. The Effects of ALR-S on Oxidative Stress in FeCl_3_-Injured HAECs

**Figure 4:**
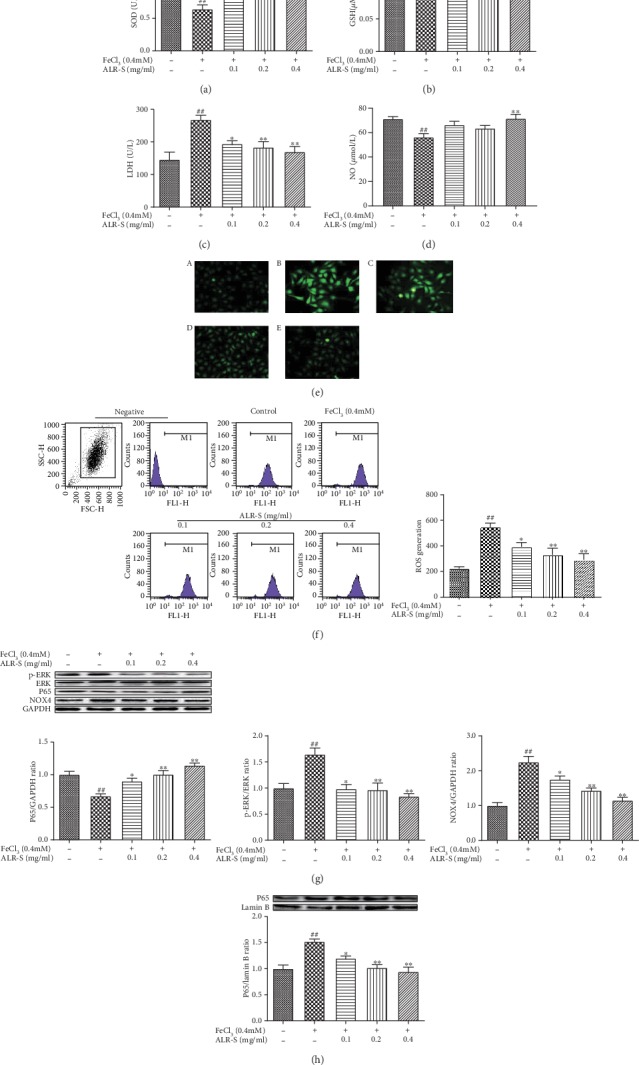
The effects of ALR-S on oxidative stress and ERK/NF-*κ*B signaling pathway in FeCl_3_-injured HAECs. HAECs were preincubated with different doses of ALR-S (0.1, 0.2, and 0.4 mg/ml) or serum-free medium for 4 h and then stimulated with 0.4 mM FeCl_3_ for 2 h. (a) SOD activity in HAECs, *n* = 6. (b) GSH activity in HAECs, *n* = 6. (c) LDH levels in culture medium of HAECs, *n* = 6. (d) NO levels in culture medium of HAECs, *n* = 6. (e) Fluorescence image of ROS in HAECs stained with DCFH-DA, *n* = 3. (f) Flow cytometry analysis of ROS in HAECs stained with DCFH-DA, *n* = 3. (g) Protein expression level of p-ERK, NOX4, and cytoplasmic P65 in HAECs, *n* = 3. (h) Protein expression level of nuclear P65 in HAECs, *n* = 3. ^#^*p* < 0.05 and ^##^*p* < 0.01 compared to the control group; ^∗^*p* < 0.05 and ^∗∗^*p* < 0.01 compared to the FeCl_3_ group.

As shown in Figure 4, compared with the control group, the FeCl_3_ treatment group showed significantly decreased GSH and SOD activities and NO release, but increased NOX4 protein expression, LDH release, and ROS generation, while ALR-S treatment reversed these changes induced by FeCl_3_.

### 3.5. The Effects of ALR-S on the ERK/NF-*κ*B Signaling Pathway in FeCl_3_-Injured HAECs

The ERK/NF-*κ*B pathway mediates dysfunction of VECs and the release of thrombosis or coagulation-related factors [29, 33, 34]. p-ERK and nuclear P65 levels were significantly increased, and cytoplasmic P65 levels were significantly decreased in the FeCl_3_-treated group compared with the control group. ALR-S treatment significantly reversed these changes induced by FeCl_3_ (Figures 4(g) and 4(h)).

### 3.6. The Effects of ALR-S and U0126 on Platelet Adhesion to FeCl_3_-Injured HAECs

**Figure 5:**
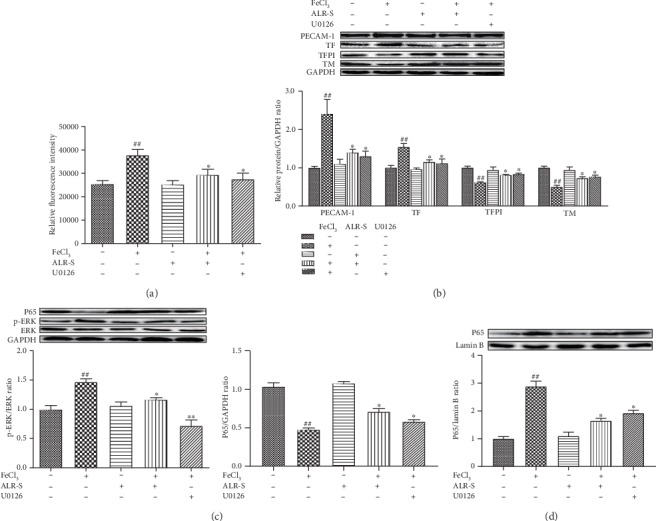
The effects of ALR-S and U0126 on platelet adhesion to FeCl_3_-injured HAECs. HAECs were preincubated with ALR-S (0.4 mg/ml), U0126, or serum-free medium for 4 h and then stimulated with 0.4 mM FeCl_3_ for 2 h. (a) The effects of ALR-S and U0126 on platelet fluorescence intensity, *n* = 3. (b) Protein expression levels of TF, TFPI, TM, and PECAM-1 in HAECs, *n* = 3. (c) Protein expression levels of p-ERK and cytoplasmic P65 in HAECs, *n* = 3. (d) Protein expression levels of nuclear P65 in HAECs, *n* = 3. ^#^*p* < 0.05 and ^##^*p* < 0.01 compared to the control group; ^∗^*p* < 0.05 and ^∗∗^*p* < 0.01 compared to the FeCl_3_ group.

U0126 is a specific inhibitor of ERK. U0126 decreased the platelet adhesion to HAECs compared to FeCl_3_ treatment alone (Figure 5(a)). U0126 also reversed the abnormal expression of TF, TFPI, TM, and PECAM-1 induced by FeCl_3_ (Figure 5(b)). Further experiments showed that U0126 significantly inhibited ERK phosphorylation and nuclear P65 levels compared to the FeCl_3_ group (Figure 5(c), 5(d)). At same time, ALR-S exhibited the same effects on the abovementioned indices with U0126 (Figure 5(a)–5(d)).

## 4. Discussion

Thrombotic disorders are one of the most common causes of morbidity, disability, and mortality in the world. Arterial thrombosis may cause ischemic stroke (IS) and ischemic heart disease (IHD), even acute myocardial infarction, which are often life-threatening. Polysaccharides, one of the most important active ingredients of traditional Chinese medicine decoction, have attracted more and more attention. ALR-S, extracted from a traditional medicinal plant, showed multiple pharmacological activities. However, the effects and mechanism of ALR-S on thrombosis have not been reported. The present study demonstrated that ALR-S effectively alleviated arterial thrombosis by rebalancing thrombotic/antithrombotic factors and inhibiting platelet adhesion. The underling mechanism is related to the inhibition of the OS and ERK/NF-*κ*B pathway, leading to VEC protection.

VECs are very important to maintain blood homeostasis. VEC dysfunction caused by various stimuli promotes thrombosis in the body. Oxidative stress is a common and important cause of endothelial dysfunction. FeCl_3_ is widely used to establish a thrombus model for scientific researches in vivo, and previous reports indicated that FeCl_3_-mediated thrombosis involves inducing VEC oxidative injury and platelet adhesion [35, 36]. Therefore, FeCl_3_-induced arterial thrombosis model and FeCl_3_-induced HAEC injury model were used, respectively, in in vivo and in vitro experiments to investigate the effects and mechanism of ALR-S on arterial thrombosis.

VECs are extensively involved in blood homeostasis by synthesis and secretion of many active substances to regulate platelet activation, coagulation, and fibrinolysis. VEC damage is an initial stage of FeCl_3_-induced thrombus formation [36]. TF is released on VEC destruction and initiates the extrinsic coagulation process, leading to fibrin clot and thrombus formation [37, 38]. TFPI, a specific inhibitor of extrinsic coagulation, inactivates TF by forming a FXa-TFPI-FVIIa-TF tetramer [39]. vWF, stored in Weibel-Palade bodies, is secreted at high levels on VEC damage to directly recruit platelets to adhere to VECs [40]. TM inhibits the fibrin clot formation and platelet activation. Plasma TM and vWF are also thought as key indicators of VEC damage. PGI_2_ and PECAM-1 are secreted on stimuli to inhibit platelet aggregation. This study showed that ALR-S significantly inhibited FeCl_3_-induced arterial thrombosis in rats by rebalancing the abovementioned thrombotic/antithrombotic factors, including vWF, TF, TXB_2_ (the metabolite of TXA_2_), 6-keto-PGF1*α* (the metabolite of PGI_2_), TM, TFPI, and PECAM-1. In vitro studies also showed that ALR-S inhibited platelet adhesion to HAECs and improved thrombosis-relative imbalance (vWF, TF, PECAM-1, 6-keto-PGF1*α*, TFPI, and TM) induced by FeCl_3_ injury in HAECs. These results suggest that ALR-S exert the antithrombotic effects mainly by protecting VECs and improving VEC dysfunction.

Endothelial OS state was examined further to investigate the mechanism of ALR-S on arterial thrombosis. OS plays an important role in the early regulation of thrombosis by activating VECs. Activated VECs then produce a large amount of ROS, leading to a vicious circle of EC dysfunction [41]. The present study showed that ALR-S significantly downregulated plasma levels of H_2_O_2_, LDH, and MDA and the expression of endothelial NOX4 and upregulated SOD activities in FeCl_3_-induced artery thrombosis in rats. These results indicated that ALR-S inhibited OS via regulating the relative enzymes, as evidenced by decreased ROS production and lipid peroxidation. In vitro studies showed that ALR-S inhibited OS in HAECs by reversing the FeCl_3_-induced changes in the production of ROS, the activities of GSH and SOD, and the expression of endothelial NOX4. These results in in vivo and in vitro investigations suggest that ALR-S exert the role of protecting VECs and improving VEC dysfunction mainly by inhibiting endothelial OS.

As previously reported, iron can induce cell oxidative damage and produce higher ROS levels to activate ERK1/2 phosphorylation. NF-*κ*B is then activated by p-ERK and translocates into the nucleus to bind to specific DNA fragments and regulate gene and protein expression [42–44], leading to EC damage, thrombosis, and other cardiovascular disease. The present study showed that ALR-S significantly downregulated the expression of p-ERK1/2 and nuclear P65, suggesting the blocking effects of ALR-S on the ERK/NF-*κ*B pathway. U0126, the accepted specific inhibitor of ERK, is frequently used to block the ERK/NF-*κ*B pathway. Here, U0126 was used for further clarifying the role of the ERK/NF-*κ*B pathway in mediating ALR-S action. As expected, U0126 demonstrated the same effects with ALR-S on FeCl_3_-injured HAECs, including inhibiting platelet adhesion and rebalancing the factors of TF, TFPI, PECAM-1, and TM. Moreover, both U0126 and ALR-S inhibited the nuclear translocation of NF-*κ*B, as evidenced by the decreased expression of nuclear P65. These results suggest that ALR-S may inhibit the ERK/NF-*κ*B pathway and then the NF-*κ*B translocation into the nucleus, leading to the downregulated expression of thrombotic factors and upregulated expression of antithrombotic factors.

## 5. Conclusion

In conclusion, our research revealed the effects and mechanism of ALR-S against arterial thrombosis in vitro and in vivo. ALR-S inhibited platelet adhesion to VECs and rebalanced thrombotic/antithrombotic factor expression and secretion by inhibiting the oxidative stress and ERK/NF-*κ*B pathway in VECs, which may lead to the VEC protection and the alleviation of arterial thrombosis. The study also indicated that the ERK/NF-*κ*B pathway may be a potential therapeutic target in arterial thrombosis and ALR-S could have a positive role in arterial thrombosis and related cardiovascular diseases.

Further studies are needed to clarify the regulatory mechanism of ALR-S on platelet adhesion and aggregation via effects on platelets and effects on the interaction of VECs and platelets in arterial thrombosis.

## Data Availability

All data used to support the findings of this study are included within the article.

## Abbreviations

ALR-S: Saccharides from Arctium lappa. L. root
FeCl_3_: Ferric chloride
HAECs: Human aortic endothelial cells
ERK: Extracellular regulated protein kinases
p-ERK: Phosphoextracellular regulated protein kinases
NF-*κ*B: Nuclear factor-kappa B
VECs: Vascular endothelium cells
ROS: Reactive oxygen species
MDA: Malondialdehyde
SOD: Superoxide dismutase
H_2_O_2_: Hydrogen peroxide
LDH: Lactate dehydrogenase
GSH: Glutathione
NO: Nitric oxide
OS: Oxidative stress
GAPDH: Glyceraldehyde-3-phosphate dehydrogenase
PGI_2_: Prostacyclin
TXA_2_: Thromboxane A_2_
TXB_2_: Thromboxane B_2_
TFPI: Tissue factor pathway inhibitor
TF: Tissue factor
6-Keto-PGF_1*α*_: 6-Keto-prostaglandin F_1*α*_
vWF: von Willebrand factor
PECAM-1: Platelet endothelial cell adhesion molecule-1
TM: Thrombomodulin
FPA: Fibrinopeptide-A
ADP: Adenosine diphosphate
NADPH: The reduced form of nicotinamide adenine dinucleotide phosphate
NOX4: NADPH oxidase 4
PRP: Platelet-rich plasma
PPP: Platelet-poor plasma
IS: Ischemic stroke
IHD: Ischemic heart disease.
